# microRNA let-7i-5p mediates the relationship between muscle fat infiltration and neck pain disability following motor vehicle collision: a preliminary study

**DOI:** 10.1038/s41598-021-82734-y

**Published:** 2021-02-04

**Authors:** James M. Elliott, Cathleen A. Rueckeis, Yue Pan, Todd B. Parrish, David M. Walton, Sarah D. Linnstaedt

**Affiliations:** 1 Faculty of Medicine and Health, The Northern Sydney Local Health District, The Kolling Institute, The University of Sydney, St. Leonards, NSW Australia; 2 Physical Therapy and Human Movement Sciences, Feinberg School of Medicine, Northwestern University, Chicago, IL USA; 3 Institute for Trauma Recovery, University of North Carolina, Campus Box #7010, Chapel Hill, NC 27599-7010 USA; 4 Department of Biostatistics, University of North Carolina, Chapel Hill, NC USA; 5 Department of Radiology, Feinberg School of Medicine, Northwestern University, Chicago, IL USA; 6 School of Physical Therapy, Western University, London, ON Canada; 7 Department of Anesthesiology, University of North Carolina, Chapel Hill, NC USA

**Keywords:** Mechanisms of disease, miRNAs, Microbiology, Molecular biology, Trauma, Neuropathic pain, Magnetic resonance imaging, Disability

## Abstract

Persistent neck-pain disability (PNPD) is common following traumatic stress exposures such as motor vehicle collision (MVC). Substantial literature indicates that fat infiltration into neck muscle (MFI) is associated with post-MVC PNPD. However, little is known about the molecular mediators underlying this association. In the current study, we assessed whether microRNA expression signatures predict PNPD and whether microRNA mediate the relationship between neck MFI and PNPD. A nested cohort of 43 individuals from a longitudinal study of MVC survivors, who provided blood (PAXgene RNA) and underwent magnetic resonance imaging (MRI), were included in the current study. Peritraumatic microRNA expression levels were quantified via small RNA sequencing, neck MFI via MRI, and PNPD via the Neck Disability Index two-weeks, three-months, and twelve-months following MVC. Repeated measures regression models were used to assess the relationship between microRNA and PNPD and to perform mediation analyses. Seventeen microRNA predicted PNPD following MVC. One microRNA, let-7i-5p, mediated the relationship between neck MFI and PNPD. Peritraumatic blood-based microRNA expression levels predict PNPD following MVC and let-7i-5p might contribute to the underlying effects of neck MFI on persistent disability. In conclusion, additional studies are needed to validate this finding.

## Introduction

While most individuals recover following traumatic stress exposures such as motor vehicle collision (MVC), a substantial subset develop adverse posttraumatic neuropsychiatric sequelae such as persistent neck pain and persistent neck-pain related disability (PNPD)^1–4^, which is typically measured by a self-report region-specific disability scale (such as the Neck Disability Index (NDI)^5^). Recent studies have identified psychosocial and demographic predictors of PNPD and co-occurring neuropsychiatric disorders^3,4,6–9^, yet little is known regarding molecular and blood-based predictors^10^. Additionally, the underlying pathogenesis that drives the transition from acute to persistent neck-pain related disability is not fully understood. This lack of understanding of biological variation in the early peritraumatic period impedes the discovery of blood-based risk biomarkers and tractable targets for preventative interventions.

Since their discovery, microRNAs have been shown to be powerful regulators of the mammalian transcriptome, play highly influential roles in disease pathogenesis, and represent a promising new class of therapeutic targets^11–13^. Additionally, microRNAs are also highly stable in blood^14–16^ and serve as promising blood-based biomarkers of disease^17^. Recent cross-sectional human cohort studies indicate that blood microRNAs are associated with PNPD related outcomes of trauma^18–20^ and prospective studies indicate that microRNAs can predict similar posttraumatic sequelae^21–23^. Such biomarker discovery has also led to the increased understanding, via back-translational and animal model studies, of the role of microRNAs in the pathogenesis of PNPD and related post-trauma outcomes^19,24–26^.

In addition to microRNAs as blood-based predictors of adverse posttraumatic outcomes, substantial research efforts have focused on understanding the contribution of skeletal muscle changes to post-trauma recovery, including how fatty infiltration of cervical muscle (MFI) influences tissue/stress injuries in the neck^27–33^. Recent longitudinal studies have demonstrated that MFI is predictive of^28^ and associated with^31–34^ PNPD, yet the molecular mechanisms mediating this association are not yet clear.

In this preliminary study, we combined advanced fat/water magnetic resonance imaging (MRI) data of neck muscle and fat composition with blood-based peritraumatic microRNA expression levels to gain insights into microRNA predictors of PNPD (per NDI % scores). In addition, we used in silico analyses to gain insight into shared pathways through which the collective set of PNPD-predictive microRNAs might influence post-trauma recovery. Finally, we assessed how microRNAs might mediate the relationship between neck MFI and PNPD development.

## Methods

All analyses were performed using biological, sociodemographic, neuroimaging, and longitudinal outcome data from a prospective observational cohort of multiethnic men and women enrolled following MVC trauma.

### Motor vehicle collision (MVC) cohort study

The details of this longitudinal observational cohort have been described previously^30^. The current study is a secondary analysis of data investigating the neuromuscular mechanisms underlying poor recovery following MVC (ClinicalTrials.gov Identifier: NCT02157038). Ninety-seven participants were recruited in the full cohort, were consented, and enrolled via an urban academic emergency medicine department and were eligible provided they both reported MVC-related neck pain (4 or > on a numeric pain rating scale) and were within the Quebec Task Force Classification category of Whiplash Associated Disorder Grade II (movement restriction with no radicular symptoms). Exclusion criteria included participant age younger than 18 or older than 65, one or more previous MVC in their lifetime, treatment for neck pain disorders in the past ten years, any nervous system disorder (e.g. stroke, Parkinson’s), metabolic system disorder (e.g. diabetes), or those who, by standard emergency medical service protocols were deemed to be at risk for multi-system trauma. The Institutional Review Board of Northwestern University, Feinberg School of Medicine granted approval (STU00090769) and all participants provided informed written consent. All methods (both in the enrollment of the MVC cohort and all subsequent sample processing and analyses) were performed in accordance with the relevant guidelines and regulations set forth by this Board. As part of this longitudinal parent study, all enrolled participants, underwent serial MRI examination at < 1 week, 2-weeks, 3-months, and 12-months post injury to quantify MFI in select bodily muscles. All participants completed a suite of questionnaires including one pertaining to neck-related disability. Only participants without any injury other than whiplash from the MVC were included in the study. Based on initial assessments, participants were screened and enrolled in the study if they did not demonstrate other comorbidities or mental health diagnoses within the last decade. For this sub-study, blood RNA samples were collected from forty-three participants at the time of enrollment (i.e. < 1 week following MVC), and all collected samples were included in the present analysis. In the nested cohort used for the current set of analyses, MRI and blood collection occurred in the same visit and on average was 4.8 ± 1.9 days following MVC trauma.

### Self-reported neck-pain disability

Self-reported neck-pain disability (i.e. how neck pain affects an individual’s ability to manage everyday life) was measured using the Neck Disability Index (NDI)^5^. This 10-item questionnaire measures a patient's self-reported neck pain disability on a scale of 0–50 via individual statements about neck pain intensity, headaches, personal care, lifting heavy objects, reading, concentrating, working, driving, sleeping, and recreation. The total score can be multiplied by 2 to produce a percentage score. Percentage scores ≥ 30% have been reported to indicate moderate/severe neck-related disability^31^ and extensive psychometric testing has indicated adequate measurement invariance to be used as a reliable indicator of self-reported PNPD over time^35^.

### Self-reported pain intensity

The Numeric Pain Rating Scale was used as a unidimensional measure of pain intensity in which the respondent selects a whole number (0–10 integers) that best reflects the intensity of their pain^36^. Higher initial pain (> 5.5/10) intensity has been associated with worse outcomes^36^.

### Self-reported levels of distress

Symptoms of distress were measured using total symptom severity score of the Posttraumatic Stress Diagnostic Scale (PDS)^37^. Higher scores (out of 51) indicate more severe symptoms. Previous work supports using the hyperarousal subscale score as a measure of distress^28,38^. Accordingly, we used the hyperarousal subscale score as a way to measure emotional-mental distress^37^.

### MRI muscle fat analysis

Muscle fat determination from magnetic resonance imaging (MRI) has been described previously^30^. All post-MVC MRI data were collected with a 3.0 T Prisma scanner (Siemens, Erlangen, Germany). A localizer scan and a T2-weighted sagittal turbo spin echo sequence was performed to determine the location of the fat–water scan. High-resolution axial 3D fat–water images of the cervical spine area were acquired using a dual-echo gradient-echo sequence (2-point Dixon, TR = 7.05 ms, TE1 = 2.46 ms, TE2 = 3.69 ms, flip angle = 12°, bandwidth = 510 Hz/pixel, FOV = 190 × 320 mm^2^, slab oversampling of 20% with 40 partitions to prevent aliasing in the inferior-superior direction, in-plane resolution = 0.7 × 0.7 mm^2^, slice thickness = 3.0 mm, number of averages = 6, acquisition time = 4 min 5 s). The scanner outputs the in- and opposed-phased data as well as the water and fat images. A 64-channel head/neck coil was used as a receiver coil to improve signal-to-noise. This scan covered the cephalad portion of C3 through the caudal portion of the C7 vertebral end plate. To address the current set of study aims, MFI was calculated using MRI data from the initial timepoint (< 1 week) only.

### Muscle water-fat quantification

Using OsiriX MD image processing software (Special limited version 2019, Pixmeo, Geneva, Switzerland; https://www.osirix-viewer.com/), regions of interest were manually drawn within the fascial borders of the multifidus-semispinalis cervicis from C4 to C7, on the co-registered fat and water images. The software obtains the mean signal intensity within each region of interest, for fat and water. The MFI (%) from 3D water-fat imaging was calculated as the mean pixel intensity of fat-only (Fat) and the mean pixel intensity of water-only (Water) images using the following equation: MFI (%) = Fat/(Fat + Water)*100^30^. MFI for the left and right multifidus-semispinalis cervicis were measured for each participant. Total MFI was defined as the mean of the left and right MFI at that level. Average of total MFI of all the cervical levels for each patient was calculated.

### RNA collection and isolation

Blood samples were collected at the time of enrollment using PAXgene RNA tubes, were incubated at room temperature for two hours, then frozen at − 80 °C until batch shipment on dry ice to the University of North Carolina at Chapel Hill. Total RNA (including microRNA) was isolated using the PAXgene blood microRNA kit (Qiagen, Germantown, MD), and RNA was stored at − 80 °C until use.

### Next-generation small RNA sequencing

Template libraries for microRNA next-generation sequencing were produced from 1.0-µg total RNA. The samples were prepped using TruSeq Small RNA library prep kits according to manufacturer's specifications (Illumina, San Diego, CA). Twelve barcoded libraries were combined per lane and sequenced on a HiSeq 2000 (Illumina). Raw sequencing reads were processed using a custom bioinformatics pipeline as described previously^22^ and were normalized using upper quartile normalization. microRNAs with an average read count of five counts or less were removed from the dataset, leaving a total of 278 microRNAs for the current analyses. Of note, blood expression levels of let-7i-5p were consistent with previous reports (sequencing reads, mean ± SD = 1208.4 ± 399.5)^39^.

### RT-qPCR validation

We used the stem-loop RT-qPCR method described by Chen et al. to quantify let-7i-5p levels in the same samples used for RNA sequencing^40^. Stem-loop RT primers and TaqMan probes for let-7i-5p and endogenous control RNU48 were obtained from ThermoFisher (Waltham, MA). MiRNA expression was quantified on a QuantStudio QS3, data uploaded to the ThermoFisher Cloud, and analyzed using the Applied Biosystems visualization portal. Relative quantitation (RQ) values were calculated via the 2-ΔΔϹt methodology using RNU48 as the normalizing control. RQ values were then compared to RNA sequencing values using Pearson’s correlation.

### Statistical analyses

Of the total 97 individuals enrolled in the parent study, the only individuals that were included in the current set of analyses were those from which an RNA PAXgene blood tube was collected (collection of these tubes commenced halfway through the MVC study, when needed funding for collection was acquired). Cohort sociodemographic characteristics were summarized using standard descriptive statistics. Generalized Estimating Equation (GEE) models including random effects were used to evaluate the association between each of the peri-traumatically expressed blood microRNAs and persistent PNPD outcomes for all time points combined (< 1-week, two weeks, three months, and one year). Models were adjusted for potential confounding by age, sex, time since the traumatic event, and participant Body Mass Index (BMI). microRNAs with a significance threshold of *p* < 0.05 and effect size of β =  ± 5 were considered significantly associated with PNPD. These thresholds were selected based previously defined thresholds^41^, sample size estimates, and post-hoc analyses of the distribution of β-coefficients such that microRNAs to the left and right of the inflection point in the distribution of coefficients were selected (i.e. β =  ± 5). The relationship between MFI and PNPD was also assessed using repeated measures mixed models adjusting for the same potential confounders as above. Statistical analyses were carried out using R 3.5.3^42^.

To explore specific microRNA transcripts that might mediate the relationship between MFI and PNPD, mediation modeling procedures were employed^43^. Mediation model analyses were performed independently for each microRNA transcript. Briefly, in step 1 of mediation analyses, we assessed the relationship between MFI and PNPD; in step 2, we assessed the relationship between MFI and microRNA transcripts; in step 3 we assessed the relationship between MFI + microRNA and PNPD. In step 2, microRNA transcripts were filtered based on a statistically significant relationship with MFI. In step 3, if the effect of MFI on PNPD was no longer significant with the addition of a microRNA transcript from step 2, that microRNA transcript was considered a mediator.

### Bioinformatics analyses

A web-based computational tool, DIANA miRPath v3.0, was used to identify molecular pathways overrepresented in predicted targeting by the seventeen differentially expressed microRNAs. DIANA miRPath uses its predictive binding algorithm, DIANA-microT-CDS, to define a list of potential targets for each microRNA, then assigns a Kyoto Encyclopedia of Genes and Genomes (KEGG) pathway^44^ rank and significance level based on the relative number of targets in that pathway. DIANA miRPath results have been validated and its predictive binding algorithm has been shown to have high concordance with actual microRNA binding. MicroRNAs that have been experimentally validated to bind to the predicted mRNA were identified using TarBase v8.0^45^.

## Results

### Cohort characteristics

Characteristics of the 43 participants who provided blood samples at enrollment are shown in Table 1. Participants were multiethnic and most were women, less than forty years old, and had a normal-to-overweight body mass index. Distress levels at the time of enrollment were low, pain was moderate-severe, and neck pain disability was moderate to severe (mean NDI = 33.7%).

**Table 1:** Baseline characteristics of study participants (n = 43).

Characteristic	
Age, years, mean (SD)	36.3 (11.4)
Women, n (%)	33 (76.7)
Ethnicity, n (%)	
African American	11 (25.6)
European American	22 (51.2)
Asian, Hispanic, Other	10 (23.2)
BMI, mean (SD)	25.7 (4.5)
Initial distress^#^, mean (SD)	11.6 (11.1)
Initial pain severity^&^, mean (SD)	4.8 (2.0)
Initial neck disability^^^, mean (SD)	33.7 (15.3)
Initial percent fatty infiltrate in neck muscle, mean (SD)	20.6 (6.0)

MVC, motor vehicle collision trauma; SD, standard deviation; ED, emergency department.

^#^Distress was measured with the hyperarousal subscale of the PDS inventory ^&^Pain severity was measured using the numeric rating scale (0–10 NRS). ^^^Neck disability was measured with the Neck Disability Index (range 0–50).

### Course of PNPD recovery over one year

**Figure 1:**
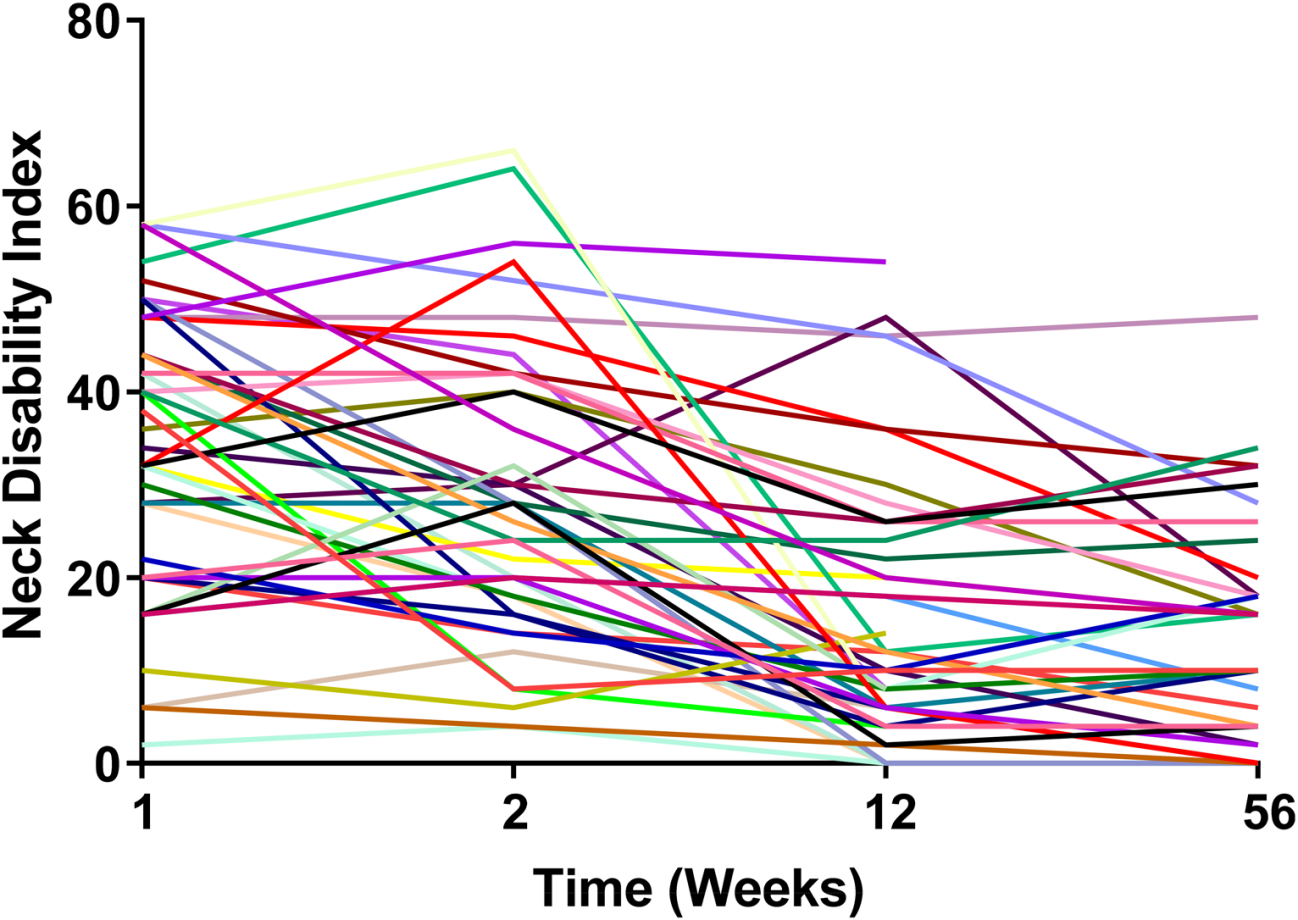
Graphical representation of neck-pain related disability index scores for each of the forty-three participants included in the current study analyses. Neck disability index questionnaires were administered at the time of enrollment following motor vehicle collision trauma (“week 1”), and at three subsequent timepoints over the course of a year (week 2, 12, and 56). Each line corresponds to a separate individual. These outcome trajectories served as the dependent variable in regression models assessing the relationship between microRNA and neck-related disability.

Neck pain related disability scores varied widely in the week following MVC (Fig. 1), with an average NDI score of 33.72 (± 15.3). Most individuals with high initial NDI scores recovered over time following MVC but a substantial number of individuals experienced persistent neck pain disability over the course of one year (Fig. 1). One-year following MVC, average neck pain related disability in this cohort was 13.8 (± 12.1). Outcome trajectories as presented in Fig. 1 served as the dependent variable in regression models assessing the relationship between microRNAs and PNPD. At the one year follow-up, 30% of the sample scored < 8% on the NDI scale indicating no substantive ongoing disability, while 12% scored ≥ 30% on the NDI scale indicating ongoing moderate to severe disability.

### Blood expression levels of seventeen microRNA predicted PNPD outcome trajectories following MVC

**Figure 2:**
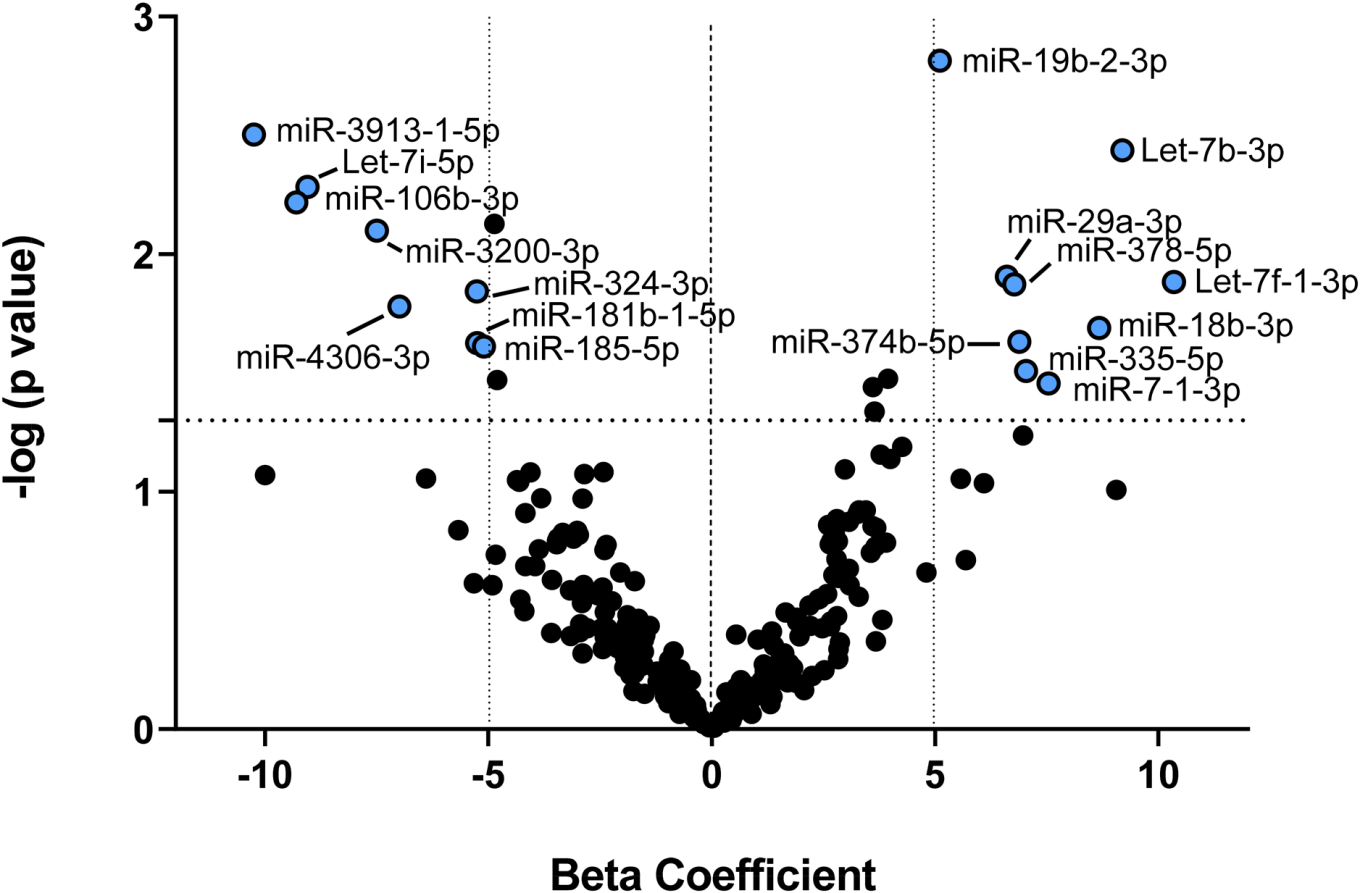
Volcano plot showing microRNA that predict persistent neck pain disability following motor vehicle collision trauma. Statistically significant microRNA were defined as having a *p*-value below 0.05 (i.e. those above the horizontal dotted line) and a magnitude of effect above and below a beta coefficient of ± 5 (i.e. to the left and right of the vertical dotted lines). Statistically significant microRNA are labeled and colored blue.

Seventeen of 278 (6%) microRNAs detected in blood samples predicted PNPD outcomes (Table 2). Thirteen of these seventeen predictive microRNAs have previously been shown to be associated with stress, pain, skeletal muscle, and/or adiposity (see ‘Previous Assoc’ column, Table 2). Among the most significantly associated microRNAs and strongest effect sizes with PNPD trajectories included miR-19b-2-3p (β = 5.11, *p* = 0.002), miR-3913–1-5p (β =  − 10.25, *p* = 0.003), let-7b-3p (β = 9.19, *p* = 0.004), and let-7i-5p (β =  − 9.05, *p* = 0.005) (Fig. 2). RT-qPCR was used to technically validate RNA sequencing results for let-7i-5p, based on subsequent analyses in the current study indicating further importance of this miRNA. Comparison of expression values for let-7i-5p via RNA sequencing and RT-qPCR showed correlation between the two methodologies (Pearson’s r = 0.53, *p* = 0.0003). Further, using the values derived from RT-qPCR, we were able to replicate the association between let-7i-5p and PNPD trajectories (β =  − 7.82, *p* = 0.009).

**Table 2:** microRNA in whole blood circulating in the early aftermath of motor vehicle collision (MVC) that predict persistent neck pain related disability trajectories following MVC trauma.

microRNA	β Coeff	S.E	*p* value^a^	Chr (Strand)^b^	Previous Assoc^c^
miR-19b-2-3p	5.11	1.57	0.002	X ( −)	S^46,47^, P^22,48^, A^47^
miR-3913–1-5p	− 10.25	3.40	0.003	12 ( −)	–
let-7b-3p	9.19	3.10	0.004	22 ( +)	S^49^, P^50^
let-7i-5p	− 9.05	3.18	0.005	12 ( +)	S^51,52^, A^53,54^
miR-106b-3p	− 9.30	3.33	0.006	7 ( −)	M^55^, A^56^
miR-3200-3p	− 7.50	2.78	0.008	22 ( +)	–
miR-29a-3p	6.61	2.60	0.012	7 ( −)	M^57^, A^58^
let-7f.-1-3p	10.34	4.11	0.013	9 ( +)	M^59^
miR-378-5p	6.77	2.70	0.013	5 ( +)	M^60^, A^61^
miR-324-3p	− 5.26	2.12	0.014	17 ( −)	S^19^, M^62^
miR-4306-3p	− 6.99	2.88	0.017	13 ( +)	–
miR-18b-3p	8.67	3.69	0.020	X ( −)	A^63^
miR-374b-5p	6.88	3.00	0.023	X ( −)	M^64^
miR-181b-1-5p	− 5.25	2.29	0.024	1 ( −)	M^65^, A^66^
miR-185-5p	− 5.10	2.24	0.024	22 ( +)	A^67^
miR-335-5p	7.03	3.22	0.031	7 ( +)	S^68^, M^69^, A^70^
miR-7–1-3p	7.54	3.54	0.035	9 ( −)	–

^a^p values were calculated using repeated measures mixed models. ^b’^Chr’ refers to the chromosome name/number from where each microRNA is transcribed and ‘strand’ refers to whether it comes from the sense ( +) or antisense ( −) strand of the genome ^c^Previous assoc = References describing a previously identified role for the microRNA in stress system biology (S), pain pathobiology (P), skeletal muscle (M), or adipose tissue (A). Coeff. = β coefficient from linear mixed models. S.E. = standard error.

### Fatty infiltration of muscle (MFI) in the neck predicts PNPD following MVC

**Figure 3:**
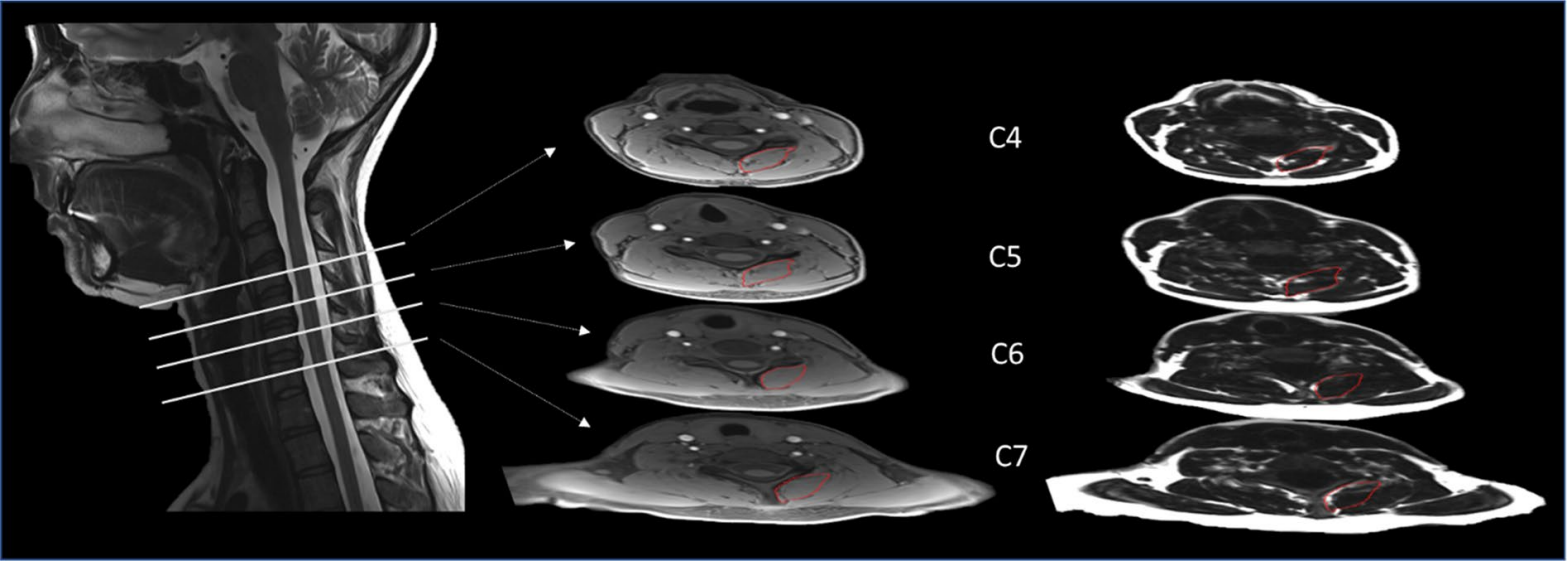
Schematic illustrating how fat infiltration into neck muscle (MFI) was measured via magnetic resonance imaging (MRI). Fat and water in the neck was measured via T2-weighted sagittal MRI of the Cervical Spine in four cross-sections (left, C4–C7). In each of these sections, water (middle) and fat (right) were quantified in the areas outlined in red (fascial borders of the multifidus-semispinalis cervicis) on both the left and right sides. The mean signal intensity for fat and water was used to quantify MFI percentage as described in the Methods. These values were calculated for each individual in the cohort and ranged from 10.4 to 38.8%.

Fatty infiltration of muscle (MFI) in the neck was measured using 3D fat/water images (Fig. 3). Using repeated measures mixed models adjusting for age, gender, time since MVC, and BMI, we identified a positive and statistically significant relationship between neck MFI and PNPD (β = 0.537, *p* = 0.0451; Table 3), indicating that higher levels of neck MFI predicted increased PNPD following MVC.

**Table 3:** The relationship between fatty infiltrates in the neck (MFI) and persistent neck pain disability in motor vehicle collision survivors examined as part of this study.

	β	S.E	t value	*p* value
Intercept	23.651	9.279	2.441	0.0162
MFI	0.537	0.265	2.026	0.0451
Time	− 0.207	0.055	− 3.754	0.0002
Gender	− 6.334	3.315	− 1.911	0.0585
Age	0.052	0.138	0.376	0.708
BMI	− 0.419	0.331	− 1.264	0.209

### Let-7i-5p mediates the relationship between neck MFI and PNPD following MVC trauma

**Figure 4:**
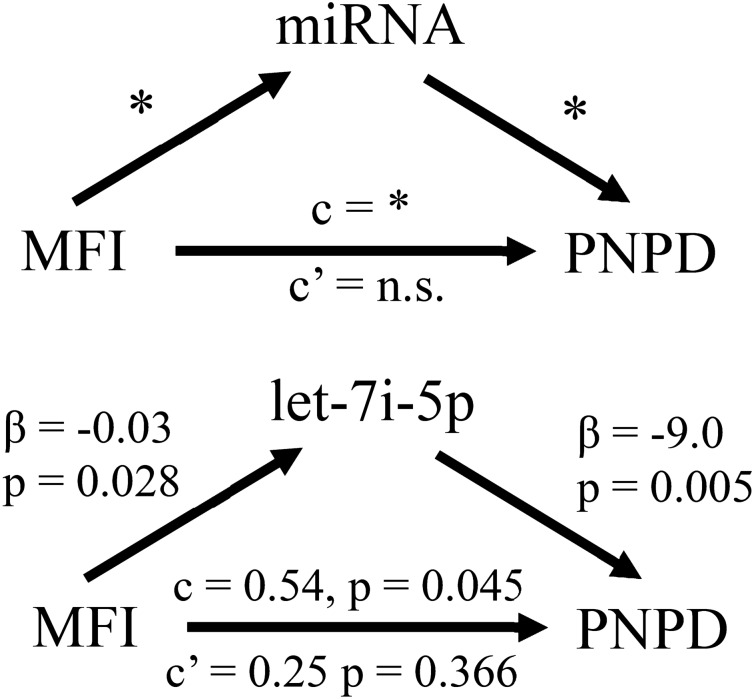
Mediation model and results of mediation analyses assessing for microRNA (miRNA) that mediate the relationship between fatty infiltrates of the neck muscle (MFI) and persistent neck pain disability (PNPD). *Top*: Schematic of the mediation model used to assess whether a particular miRNA or set of miRNA transcripts might mediate the relationship between MFI and PNPD development following motor vehicle collision trauma. “c” refers to the total effect in the unmediated model and “c’ ” refers to the direct effect in the mediation model. We assessed for complete mediation (versus partial) thus were interested in miRNA transcripts that caused MFI to no longer affect PNPD (i.e. “n.s.”) upon addition of the miRNA transcript to the model. *Bottom*: MFI relationship with let-7i-5p (leftmost arrow), let-7i-5p relationship with PNPD (rightmost arrow), and MFI relationship with PNPD with and without mediation by miRNA (horizontal arrow). Mediation was assessed for all miRNA identified in Table 2 but let-7i-5p was the only statistically significant mediator identified.

Of the seventeen microRNAs identified to predict PNPD (Table 2 and Fig. 2), let-7i-5p was the only microRNA that mediated the relationship between neck MFI and PNPD. Results of this mediation analysis are shown in Fig. 4. These results indicate that MFI is statistically significantly associated with let-7i-5p (β =  − 0.03, *p* = 0.028) and that with the addition of let-7i-5p in the model assessing the relationship between neck MFI and PNPD (Table 3, described above), MFI is no longer a statistically significant predictor of PNPD (β = 0.250, *p* = 0.366). Such results indicate full mediation by let-7i-5p.

### Evaluation of biologic pathways targeted by PNPD-associated microRNAs

Using DIANA miRPath v3.0^71^, we assessed for molecular pathways (KEGG pathways^44^) overrepresented in predicted targeting by the seventeen microRNAs that predict PNPD. The pathways with the highest number of gene transcripts targeted by the seventeen microRNAs in Table 2 (i.e., most statistically significant enrichment) out of ~ 450 KEGG pathways that DIANA miRPath queries are shown in Table 4. These pathways include the ECM receptor interaction pathway (*p* = 1.0 × 10^−325^), the TGF-β signaling pathway (*p* = 1.40 × 10^−6^), Amphetamine addiction (*p* = 1.8 × 10^−5^) and Axon guidance pathway (*p* = 1.8 × 10^−4^). Let-7i-5p is a predicted regulator of the three most statistically significant KEGG pathways presented in Table 4. Substantial evidence supports an important role for these pathways in mediating physiologic responses to stress and the pathogenesis of pain and posttraumatic PNPD^72–78^.

**Table 4:** DIANA miRPath predicted KEGG pathways enriched in targeting by the seventeen significant microRNA expressed in the early aftermath of motor vehicle collision and that predict persistent neck pain related disability over the course of one year.

KEGG pathway	*p* value	microRNA	Predicted targets
ECM-receptor interaction	< 1.0 × 10^−325^	let-7i-5p miR-3200-3p miR-29a-3p miR-18b-3p	COL27A1*, COL3A1*, COL1A1*, COL1A2*, ITGA7*, COL4A6*, COL5A2*, COL4A1*, ITGB1, COL4A5, COL24A1, COL2A1, COL4A2*,COL5A1*, COL4A3, COL4A4, LAMC1*, COL11A1, COL6A3, LAMA2, TNN, COL5A3
Signaling pathways regulating pluripotency of stem cells	1.4 × 10^−9^	let-7i-5p let-7b-3p let-7f.-1-3p miR-374b-5p miR-7–1-3p	DVL3*, NRAS*, HOXB1*, ACVR1B*, HAND1*, SMARCAD1*, IGF1R*, FZD3*, FZD4*, RIF1*, SKIL*, ACVR2A*, ACVR1C*, IGF1*, PCGF3*, WNT9A*, BMI1, FZD7, JARID2, TCF3, GSK3B, WNT16, FZD5, ID2*, KAT6A, PAX6, SMAD2, INHBB, PIK3CB, REST*, WNT5A, BMPR1B, INHBA, WNT3, ZFHX3*, ID4, WNT5B, KRAS, ACVR1, ACVR2B*, PCGF5, JAK2, SMAD4, AXIN2, LIFR, ZIC3, SMAD5*, ID1, FGF2, BMP2*, IL6ST, ISL1, FGFR2, SOX2, JAK1*, BMPR2*, COMMD3-BMI1
Amoebiasis	2.0 × 10^−7^	miR-29a-3p let-7i-5p	ARG2*, COL27A1*, COL3A1*, CASP3*, COL1A1*, COL1A2*, COL4A6*, IL10*, COL5A2*, COL4A1*, COL4A5, SERPINB9*, COL2A1, COL4A2*, COL5A1*, COL4A3, PIK3R1, COL4A4, LAMC1*, COL11A1, LAMA2, COL5A3, PIK3R2
Lysine degradation	6.3 × 10^−7^	miR-29a-3p miR-324-3p miR-3200-3p miR-374b-5p miR-7–1-3p	WHSC1L1*, SETD1B*, SETDB2, PLOD2*, SETD2, NSD1, ASH1L*, SETDB1, PHYKPL, DOT1L, WHSC1, SUV420H2*, KMT2C, SETD1A*
TGF-beta signaling pathway	3.0 × 10^−6^	let-7b-3p miR-374b-5p miR-3913-5p let-7f.-1-3p miR-7–1-3p	FST, TGFBR1*, ID2*, ROCK1, SMAD2, SMAD6, INHBB, SMAD9, THBS1*, SMURF2, BMPR1B, CUL1*, INHBA, CDKN2B, ID4, RHOA, ACVR1, SKP1, ACVR2B*, ZFYVE16, DCN, SMAD4, E2F5*, SMAD5*, ID1, ACVR2A, GDF6, BMP2*, TFDP1, SP1*, ACVR1C*, LTBP1, NOG, CREBBP, BMPR2*
Amphetamine addiction	1.8 × 10^−5^	let-7f.-1-3p miR-181b-5p miR-7–1-3p miR-18b-3p let-7b-3p	CAMK2D*, PRKCA, ATF2*, CALM1*, CREB5*, PPP3R1, DRD1, SLC18A2, GRIA1, CALM2, CREB1, GRIA2, CAMK2A*, PPP3CA, MAOA, PRKCB*, CAMK2B, GRIA4, GRIN1, GRIN2A, PRKACB, PPP1CB, GRIA3, GRIN2B*
Thyroid hormone synthesis	9.2 × 10^−5^	miR-324-3p miR-19b-3p	ADCY1, ATF2*, ADCY7, SLC26A4, ATP1A2, LRP2, ITPR1, PRKX, GNAQ, CREB3L2*, TG, ADCY9*
Axon guidance	1.8 × 10^−4^	miR-185-5p let-7b-3p let-7f.-1-3p miR-7–1-3p miR-4306-3p	EFNB2, SEMA6A, PLXNA2, GSK3B, EPHB2, MET*, ROCK1, CXCR4, PAK2, ARHGEF12*, EPHA5, PAK7*, ROBO2, PPP3R1, SEMA3C, RHOA, KRAS, PAK3, FYN, EFNA5, PAK1, PPP3CA, RASA1*, SLIT2, DPYSL2, SLIT1, NFATC2, UNC5C, CFL2*, SEMA4C*, CDC42, PAK6, EFNB1, PLXNC1*, NTN4, SEMA6D, SLIT3, EPHA4*, NFATC3*, GNAI1*
Glioma	3.4 × 10^−4^	miR-29a-3p miR-181b-5p miR-7–1-3p	CAMK2D*, BRAF*, PRKCA, NRAS*, CALM3*, CALM1*, PIK3CB, IGF1R*, KRAS, CDK6*, CALM2, CAMK2A*, AKT2, PIK3R3, CCND1*, E2F3*, PDGFB*, PIK3R1, SOS1, PRKCB*, IGF1, AKT3, CAMK2B, PTEN, EGF, PDGFA, PIK3R2
Arrhythmogenic right ventricular cardiomyopathy	4.2 × 10^−4^	let-7b-3p miR-374b-5p miR-7–1-3p let-7f.-1-3p	DSC2, ITGB8, CACNB4, TCF7L2*, ITGA1, PKP2, ITGAV*, DMD*, SLC8A1, ITGA2, DSG2*, CTNNA3, GJA1, CACNA2D1, ATP2A2*, ITGA6, JUP

*Denotes mRNA that bind to let-7i-5p, as identified by TarBase v8.0^45^.

## Discussion

Currently, little is known about the molecular mediators predicting PNPD following traumatic stress exposures such as MVC. Additionally, little is known about whether molecular regulatory events mediate the relationship between fatty infiltrates in neck muscle, a validated predictor of PNPD^28,32^, and persistent neck-pain disability. In this preliminary proof-of-concept study, we combined blood-based microRNA expression data with MFI data to gain increased understanding of the interplay between physiological and molecular biological influences on the development of PNPD following MVC. We first identified seventeen microRNAs that predict PNPD and identified biological pathways in which these microRNAs are involved. We then provided a small-sample validation of the association between neck MFI and PNPD, and finally, we discovered preliminary evidence that let-7i-5p mediates the relationship between fatty infiltrates in neck muscle and PNPD following MVC.

A number of the microRNAs identified as predictors of neck-pain disability in the current study have been shown previously to be associated with both neuropsychiatric disorders that are related to neck-pain disability and biological processes involved in the pathogenesis of adverse posttraumatic outcomes. For instance, miR-19b-3p has been shown to predict persistent widespread pain and posttraumatic stress symptoms following MVC and sexual assault traumas^22^ and has been shown to be associated with other types of pain, anxiety, and physiological distress^19,23,46,48,79^. miR-106-3p has been shown to be involved in neuronal repair following injury^80^, is affected by physiological stress in animals and humans^81^, and is a negative regulator of adipocyte differentiation^56^. Let-7 microRNAs including let-7i-5p and let-7b-3p have been shown to be differentially expressed after stress exposure^51,52,81^, and multiple members of the let-7 family have been shown to be associated with chronic pain^50,82–85^. Consistent with our findings indicating that let-7i-5p mediates the relationship between fatty infiltrates in neck muscle and neck-pain disability, let-7i-5p has been shown to influence adipocyte function in both mice and humans^53^. Future studies are needed to better understand the contribution of let-7i-5p and other microRNAs to PNPD following trauma exposure and to identify whether let-7i-5p might play a role in the underlying molecular pathogenesis connecting fatty infiltration of neck muscle to the development of PNPD.

To gain additional insight into the potential influence of the seventeen identified microRNAs on the persistence of neck-pain disability following MVC, we performed biological pathway enrichment analyses. Through these in silico analyses, “extracellular matrix (ECM) receptor interaction” was the most highly enriched KEGG pathway. Components of this pathway have previously been shown to play a role in pain persistence and related disability through its effect on intervertebral disc stability (i.e. degradation of ECM by inflammatory mediators leads to spinal instability and subsequent pain^72^) and hippocampal plasticity^78^, and through the generation and transmission of nociceptive signals^77^. Additional biological pathways identified through pathway enrichment analyses included the TGF-β signaling pathway, the amphetamine addiction pathway and the axon guidance pathway. Each of these pathways have been shown to influence persistent pain and/or PNPD previously^72–78^. Of note, despite not being identified via in silico pathway analyses, it was interesting to find via a structured literature search that over half of the seventeen microRNAs identified as predictors of neck-pain disability following MVC have previously been shown to be associated with adipose tissue biology (see Table 2). Such enrichment in adipose associated microRNAs further (yet indirectly) supports the reproducible finding that fatty muscle tissue might contribute to PNPD following trauma exposure. It also suggests that microRNAs likely play a contributing role in the underlying biology of recovery versus persistent disability following MVC.

The strengths of this study include the use of a longitudinal cohort study design, paired microRNA and MRI data, and the use of mediation analyses to determine whether microRNAs transmit the effect between our independent and dependent variables. The longitudinal study design in which we measured both microRNA expression levels and fatty infiltrates of muscle in the early peritraumatic period enabled the preliminary identification of biological predictors of a persistent posttraumatic outcome. Such type of discovery is currently in its infancy but holds promise for identifying susceptibility/risk biomarkers of at-risk individuals^10^. This study design also enables the discovery of promising candidates for preventative therapeutics. The limitations of this study include the small sample size, the use of a cohort with predominately women, the lack of adjustment for medication use before, at the time of, or after MVC, and the lack of external replication of microRNA findings. Therefore, the generalizability of our results to additional populations is currently unknown. Future studies should validate this proof-of-concept work in larger and more diverse cohorts.

In conclusion, in this preliminary study, we identified a set of microRNAs that predict PNPD following MVC trauma. While modest in size and scope, these findings demonstrate promise for future large-scale work that could identify blood-based risk biomarkers and therapeutic targets for preventive intervention. Through such work, we can hope to decrease the global burden of adverse posttraumatic outcomes such as persistent neck-pain disability.
